# Role Models of Aging among Older Men: Strategies for Facilitating Change and Implications for Health Promotion

**DOI:** 10.3390/sports11030055

**Published:** 2023-02-28

**Authors:** Jordan Deneau, Rylee A. Dionigi, Paula M. van Wyk, Sean Horton

**Affiliations:** 1 Temerty Faculty of Medicine, University of Toronto, Toronto, ON M5S1A8, Canada; 2 Department of Kinesiology, Faculty of Human Kinetics, University of Windsor, Windsor, ON N9B2Z5, Canada; 3 School of Allied Health, Exercise & Sports Sciences, Faculty of Science & Health, Charles Sturt University, Port Macquarie, NSW 2444, Australia

**Keywords:** prevention, healthy lifestyle, physical activity, exercise, wellness, aging, sport

## Abstract

Understanding later-life role model choice and motivations, particularly for older men in sport, exercise, and health contexts, is complex and heterogenous, making it difficult for health and exercise promotion initiatives. This qualitative study examined: (1) whether older men have aging role models, and if so, their characteristics; and (2) older men’s reasons for role model choice, or lack thereof, and how role models can influence meaningful change in perceptions and practices associated with aging, sport, exercise, and health. Through in-depth interviews and photo-elicitation with 19 Canadian men aged 75 years and over, thematic analysis determined two key themes: *Role model choice,* and *Processes of role models facilitating change.* Four key strategies for role models facilitating change in older men were determined: *elite (biomedical) transcendence; valued exemplary endeavours; alliance connections*; and *disconnect and caveats*. Ultimately, while promoting the biomedical achievements of role models may resonate with many older men, when applied too closely in sport or exercise contexts (e.g., using Masters athletes as role models), there is potential for unrealistic standards and overmedicalization that could miss uncovering the latent importance that older men place on the diverse experiences and perspectives of aging that go beyond traditional masculine ideals.

## 1. Introduction

Within Canada, older adults represent the fastest growing age cohort [1] and simultaneously the least physically active [2]. These observations are also consistent with worldwide trends [3,4]. Many older adults do not experience the physical, psychological, social, and aging-related benefits that can be obtained through engagement (the terms engagement, participation, and involvement are used interchangeably) with sport, exercise, and physical activity [5,6]. Strategies to promote active aging (e.g., engagement with physically active pursuits) are abundant in media and policy [7], alongside the multitude of reasons older adults do not or cannot participate (e.g., physical limitations, accessibility constraints, and lack of interest [8,9,10]. Although still within its infancy, an emerging body of evidence suggests that later-life role models may be useful in promoting physical activity and exercise rehabilitation adherence among older adults [11,12,13]. The purpose of this study was to examine the extent to and the ways in which role models can facilitate participation in sport, physical activity, and exercise and cultivate positive perceptions of aging among older men. That is, a group who is typically less likely to seek help for their health and tend to be hard-to-reach in terms of involvement in formal public health promotion and disease prevention initiatives, for possible reasons related to masculine ideals of independence, solitude and/or stoicism, especially as they age (see [9,14,15,16,17]). A greater understanding of gender-sensitive health promotion strategies is necessary for all genders, yet there is very little research in this area focused exclusively on men aged 75 years and over [9,18].

### 1.1. Aging, Sport, and Physical Activity

Gerontological research and practice are predominantly underpinned by a biomedical focus on aging, with aging being framed as a process of inevitable physiological deterioration in need of intervention [19]. While the biomedical focus of aging remains essential, the overmedicalization of aging, particularly within Western societies, has been criticized for rigidly framing the aging experience, failing to account for heterogeneity in later life and plausibly contributing to widespread negative views and stereotypes of aging [20,21,22]. For example, Rowe and Kahn’s [23] Model of Successful Aging tends to be the most popularized conceptualization of ‘successful aging’ (SA), which argues that to age successfully one must attain the following three standards: avoid disease and disability, maintain high cognitive and physical functioning, and stay actively engaged with life.

This well-known concept of SA has been critiqued in the literature because it places the onus of ‘aging successfully’ on the individual, which positions older individuals as the ones to blame for failing to take responsibility for their health and thereby burdening the healthcare system, despite most age-related diseases and disabilities being random and largely uncontrollable [20,21,24]. Within this context, participation in sport and physical activity across the lifespan is typically promoted in health promotion policies and initiatives as a panacea in combatting age-related decline, disease, and disability [21,25,26,27]. Correspondingly, Masters athletes (who compete in sport at an older age) are commonly regarded in exercise sciences and health promotion discourses as exemplars or role models of successful aging [28], in a societal context where fitness and health have now become an essential part of an aging identity [19,21,29]. However, in reality, older adults, including older Masters athletes, tend to hold diverse perspectives and beliefs regarding what constitutes SA to them [28,30,31,32], and the extent to which physical activity may facilitate personal SA criteria varies greatly [18,28,33].

Masters athletes are individuals who regularly compete in and train for organized sport at regional, national, and international events [34]. Masters sport provides a competition opportunity for those who are outside of the typical ages considered conducive to peak performance for a given sport, such as over 35 years for track and field events [16,28,34]. Despite older Masters athletes being held up as exemplars of successful aging [28], the research on elite older athletes as potential role models is mixed [11,12,35], as discussed in more detail below. Therefore, it is important to approach aging, sport, and physical activity research with an awareness that older adults have various aging experiences and may be influenced by role models in unique ways, which ultimately has implications for health promotion strategies.

### 1.2. Later Life Role Models and Theories of Behaviour Change

Consistent with Bandura’s social cognitive theory [36,37] and self-efficacy theory [38], it is possible that having later-life role models may foster a positive attitude and behaviour change pertaining to aging perceptions and physical activity engagement. Bandura [37] identified four principal sources of self-efficacy (i.e., one’s belief in their ability to perform a desired behaviour): performance attainment, verbal persuasion, physiological state, and vicarious experience (or modelling). Vicarious experiences (i.e., role modelling) can increase self-efficacy in observers because they believe that they can master comparable tasks [37]. This was evident among older people (65–72 years; six women, four men) in a 12-week moderate–high intensity resistance training program by Dionigi [39,40], where participants expressed enhanced psychological well-being as an outcome of the exercise program when fellow participants acted as role models. That is, increased self-efficacy in using the exercise equipment and an attempt to lift more weights or perform more repetitions (after seeing their similarly aged peers role model it successfully) were key vicarious mechanisms underlying the health benefits they gained from the program [39,40]. The reason for such an increase in self-efficacy in this context was because the amount of uncertainty people have about their capabilities (e.g., the participants were new to resistance training) and the criteria by which ability is judged (e.g., the amount of resistance on the exercise machine) are two “conditions under which self-efficacy appraisals are especially sensitive to vicarious information” ([37], p. 399). Therefore, role models in an exercise setting are key sources for behaviour change and psychological health benefits among older people.

Role models, generally, tend to be exemplary individuals who can provide a source of identification, teaching, and motivation to individuals or groups in the attainment of their goals [41]. Examples in which role models have shown to be beneficial (e.g., in terms of health outcomes, academic motivation, career achievement) typically include those in educational and occupational contexts, which are often focused on younger cohorts (e.g., [42,43,44]). Very little systematic research has explored role models of aging for older men, despite older men being targeted in exercise promotion and interventions. There is speculation and preliminary evidence that later-life role models could lead to more positive views of aging and increased engagement in active leisure [45,46]. Levy’s [47] self-embodiment theory argued, from a psychosocial approach to aging, that one can vicariously develop positive self-perceptions of aging through role models, which may ultimately lead to beneficial self-fulfilling prophecies such as increased engagement in social, sporting, and physical activities.

A review on stereotypes of aging and their effects on the health of older adults by Dionigi [48] found that both positive and negative stereotypes of aging (and similarly, positive and negative role models of aging) can concurrently facilitate and constrain the practices, performance, decisions, perspectives, and, consequently, overall health of an older person. For example, Horton et al.’s [12] exploratory qualitative work on seniors’ perceptions and stereotypes of aging found that older adults tend to have an exemplar of someone in their lives who they believed aged well, who often represented a high quality of life in old age and their practices contrasted negative stereotypes of aging, such as later-life primarily as a time of ill-health, decline, and inactivity. Mentioned role models tended to be close family or friends who were 10–20 years older than participants. Subsequent work by Horton et al. [11] suggested that older women’s attitudes toward health-related role models differed based on participants’ levels of physical activity involvement. That is, *highly active* older women who were interviewed tended to have *negative* role models who exemplified poor or compromised health, and they were inspired to change their health-related behaviour to avoid the same negative health consequences as these individuals. On the other hand, *moderately active to inactive* older women tended to have more *positive* role models who exemplified good health, and these women were inspired to change their health-related behaviour to achieve similar health as these individuals. These findings are, in part, explained by the concept of regulatory focus, which suggests that individuals possess a health promotion or prevention orientation when looking to others for comparison [49]. Lockwood, Chasteen, and Wong [50] found that, as individuals age, their focus on role models tends to become more prevention- or ‘loss-focused’ rather than solely promotion- or ‘gain-focused’, compared to younger cohorts. Essentially, older adults may be motivated to change their health-related attitudes and behaviours to be more, or possibly less, like someone else in terms of how their role models aged and of their lifestyle practices, such as participation in sport and exercise. Overall, findings on later life role models presented thus far allude to the complexity and heterogeneity in the current understanding of role model choice and motivations, and more of this research that focuses on older men is needed.

The utility of aging role models has received little attention in the literature, particularly from a qualitative perspective, despite the influence that role models can have on older people’s health-related choices and behaviour [48]. Some studies have suggested that elite older athletes as potential role models may represent unrealistic standards for many older adults and may ultimately discourage engagement in physical activity [35]. On the other hand, Horton et al. [11,12] showed that the influence of role models is not a straightforward process, with role model choice being dependent upon an individual’s current levels and beliefs about physical activity, as well as gender, as detailed above, with some active older adults perceiving older Masters athletes as role models that could inspire personal change. Masters athletes and Masters sport are often portrayed in the media as inspiration for physically active aging (e.g., [51]), yet it appears that this strategy, while potentially effective at encouraging change for some, particularly young people, is neither applicable nor effective for everyone [52].

Furthermore, Jopp et al. [13] followed up on the work by Horton et al. [11,12] via interviewing adults across different age cohorts (range of 18–99 years of age) and investigating the characteristics of and reasons for personal aging role models, as well as their associations with negative views on aging. Role models chosen were predominantly family members, were gender-matched, and were most frequently chosen for health, activity engagement, and social reasons [13]. Notably, these three most frequently mentioned reasons are consistent with the three components of Rowe and Kahn’s [23] successful aging model outlined above. However, additional, less-frequently mentioned reasons go beyond the biomedical realm, such as attitudes toward life and finding meaning in life, suggesting that, while perceived biomedical attainments are an important part of aging, this process is likely more intricate and multifaceted for many older adults. For example, individuals in Jopp et al.’s study who selected a family member as a role model tended to mention a greater number of reasons for their role model choice in comparison to persons who selected non-family members (e.g., acquaintances, public figures). Additional reasons for role model choice were ultimately associated with less negative views on aging, when controlled for age, gender, and subjective health. Accordingly, Jopp et al. [13] speculated that having an in-depth, close connection with a family member role model may provide a more proximal, realistic, elaborate, and directly engaging exemplar of what is possible in one’s later life.

Similar to previous findings (e.g., [53]), conclusions by Jopp et al. [13] indicated that role models who can inspire differentiated concepts of aging successfully, beyond solely biomedical reasoning, may lead to more positive views on one’s own aging process. Consistent with stereotype embodiment theory [47], this outcome of a positive change in the perception of aging may lead to self-fulfilling prophecies such as health-related behaviour change (e.g., engagement in sporting, exercise, or other physical activities) and cognitive adjustment (e.g., coping) that promote aging well. Therefore, preliminary findings indicate that there is potential for aging role models to serve a key, positive, and influential function in the lives of older adults. As a result, the effectiveness of this relationship is likely highly contingent on the right match between role model and mentee characteristics and preferences.

Given the apparent complexity and novelty regarding role models for aging, exploratory qualitative research is a useful approach to deepen the understanding on this topic that ultimately aims to improve the later life experiences of our rapidly aging population (see [34,54]). There is also a need for research focused exclusively on men 75 years of age and over given the disproportionate growth of the population aged over 75 years and how this cohort might have different needs to those between 60 and 75 years and women who are aged 75 years and over [9]. By focusing on men 75 years of age and older, this study builds on and allows direct comparisons to be made to the qualitative work by Horton et al. [11] on role models and perspectives on Masters athletes among women aged 75 and over who varied in their levels of physical activity involvement.

## 2. Research Aims

This study is part of a larger project on aging and physical activity that aimed to inform health promotion initiatives with the perspectives and experiences of older adults. The current qualitative study examined: (1) whether older men have aging role models, and if so, their characteristics; and (2) older men’s reasons for role model choice, or lack thereof, and how role models can influence meaningful change in perceptions and practices associated with aging, sport, exercise, and health. This study was cleared by the lead author’s university research ethics board, and all participants had the capacity to understand study information, provide informed consent, and freely choose to participate.

## 3. Methodology

### 3.1. Qualitative Approach

This study utilized an exploratory, qualitative approach within the interpretive paradigm [55], which was positioned within a larger project informed by Heidegger’s hermeneutic phenomenology [56]. Accordingly, we sought to illuminate the essence and meanings of participants’ lived experiences [57,58] and situate these within the broader socio-cultural context and understandings of aging and interpret them using theories of behaviour change, such as those outlined above by Bandura [36,37,38] and Levy [47]. Ultimately, to deepen the understanding of older men’s experiences and perceptions pertaining to personal aging role models, we employed a pluralistic approach to data collection and analysis by combining multiple qualitative methods (i.e., semi-structured interviews, photo-elicitation, thematic analysis [59,60]).

### 3.2. Participants

Purposive sampling (see [58]) was used to recruit 19 participants according to several key variables, including age (75 years of age and older), gender (males), language (ability to speak English), place of residence (Southwestern Ontario), and level of physical activity involvement (active, inactive, and active with assistance, as defined below). See Table 1 for a summary of participant characteristics. The primary distinguishing focus between participants, and the impetus for delineating participants into separate groups for initial stages of analysis, was the type and amount of physical activity that participants engaged in, which was guided by Canadian government physical activity guidelines for older adults [61]. We determined group delineation by screening participants prior to interviews via demographic questionnaires that included self-report responses about how many days per week they engaged in exercise, how long exercise sessions lasted, and the type of exercise engaged in. Based on recommendations (e.g., [62]) and previous similar qualitative work (e.g., [33]), we initially aimed to recruit five to seven participants per physical activity group to achieve data saturation within each group.

Specifically, men in the *active* group (n = 6) participated in moderate- to high-intensity physical activity (e.g., running, brisk walking, bicycling, swimming, and strength training) for at least 150 min every week on average; men in the *inactive* (or low activity) group (n = 6) participated in less than 150 min of moderate- to high-intensity physical activity every week on average; men in the active with *assistance* group (n = 7) participated in moderate-intensity rehabilitative physical activity on a regular basis but could not otherwise perform related activities independently without health professional supervision due to at least one chronic health condition. Individuals in the latter group were recruited from a formal exercise rehabilitation program, and the defining feature of this group was not necessarily their amount of physical activity engagement, but the type of engagement, for which mere association with the rehabilitation program deemed these individuals a part of a distinctive group for study. Chronic health conditions and comorbidities within the sample were predominately physical in nature and varied within this group (e.g., cardiovascular disease, motor system disorders, musculoskeletal injuries). The sample was largely middle-class and Caucasian, with varying levels of education (see Table 1).

Participants were delineated into physical activity groups in this manner to extend previous research that noted differences in physical activity perspectives among older women, 75 years and over, who varied in their levels of physical activity involvement [33]. By exploring the perspectives of males in this context and by including a distinct exercise rehabilitation group, we aimed to address knowledge gaps that would be salient to health promotion initiatives concerned with a heterogeneous aging population. Participants provided written consent prior to participating in this project, which was cleared by a Canadian University Research Ethics Board.

### 3.3. Data Collection

Each participant completed a single data collection session with the first author in-person, commencing with a semi-structured interview and culminating in one photo-elicitation exercise. Each session took place a year prior to the emergence of the global COVID-19 pandemic and generally lasted 60–90 min, with approximately one quarter of each session focusing on the photo-elicitation aspect. Sessions were audiotaped with a digital voice recorder and subsequently transcribed verbatim by the first author to optimize familiarization and immersion with the data. All participants were assigned identification codes at the transcription stage and pseudonyms during manuscript preparation for anonymity.

The semi-structured interview guide used open-ended questions and associated prompts (see [58]) designed to elicit participants’ experiences and perspectives on aging role models and Masters athletes. Sample questions and prompts included: Tell me about someone who represents successful aging to you. Why does this person represent successful aging to you? To what extent does the role model influence your health-related attitudes and behaviours? What are your thoughts on competitive sports for older adults? The semi-structured format gave the interviewer the flexibility to probe participant responses for further detail when appropriate.

Photo-elicitation involves using visual mediums, provided by the researcher or informant, to evoke verbal discussion and create data that may add different layers of meaning and information to that gathered from conventional qualitative interviews [63,64]. This technique may produce alternative perspectives by engaging parts of human consciousness that are specific to accessing and interpreting symbolic representations within images [65]. For the purposes of the current study, participants were invited to engage in a photo-elicitation exercise for which images were provided to generate discussion relevant to aging, physical activity in later life, and role models. The research team curated a collection of 16 images of older men, or groups of older men, that represented a wide range of leisure pursuits that ranged from highly active to sedentary and varied from individual to collective in nature. Images included competitive running, ice hockey, resistance training in a gym, soccer, golf, tennis, basketball, bowling, gardening, spending time with grandchildren, playing cards with others, watching sports on television with others, playing the guitar, reading a book, driving a car, and taking a nap. All of the images were laid out in a random orientation on a table and participants were asked to select the photos, if any, that they felt most closely represent who they would identify as an aging role model and to elaborate on their choice. There were no restrictions placed on how many images participants could select. Most participants selected one image to discuss. Finally, we asked participants to comment on their views of high-level competitive athletes and sports to ascertain the plausibility of Masters athletes and Masters sporting competitions as inspirations (or otherwise) for health-related attitude and behaviour change in later life.

### 3.4. Data Analysis and Interpretation

Thematic analysis along with past research and theories were used to understand and interpret shared meanings across our pluralistic data set. Data collection and analysis were intertwined such that initial analytical thoughts and ideas were pondered and formulated at the same time as interviews and transcript immersion, as is common in qualitative research [58]. This allowed for the connection of emerging patterns, for informing the exploration of topics of interest in subsequent interviews, and for monitoring data saturation. The analysis of the photo-elicitation data set occurred simultaneously with that of the traditional interview data. Thematic analysis allowed for the flexibility of understanding meanings across a pluralistic dataset, such that there was an amalgamated analysis and interpretation of interviews and photo-elicitation [59,66].

The first author initially conducted standard coding and comparison procedures via inductive and deductive means [58,67]. This involved identifying meaning units (i.e., codes) first within physical activity groups, followed by connecting and comparing higher-order themes through axial coding across groups (see [58]). Similarities and differences in meaning units were cross-referenced across datasets, which resulted in salient nuances as reported in the findings. This triangulation strategy contributed to the rigour and trustworthiness of the data [58]. Further strategies to enhance credibility included analyst triangulation (i.e., using multiple experienced qualitative researchers to interpret findings). For example, once initial codes were generated, the co-authors acted as critical peers in an iterative debriefing process of challenging, creating, and redefining codes across several rounds. Moreover, consistent with principles of hermeneutic phenomenology [56], codes were contemplated and interpreted within several relevant contexts (e.g., sociocultural, political, and historical) and in relation to previous similar work (such as [11,33]). Additionally, relevant theories (e.g., Bandura’s Social Cognitive Theory and Self-Efficacy Theory and Levy’s Stereotype Embodiment Theory, described above) were used to guide our interpretations and deepen our understanding of the relationships and themes within the data.

## 4. Results

Participants’ perspectives on how role models may influence their health-related attitudes and behaviours demonstrated both consistencies and differences within and across activity groups, although the results are provided with the participant groupings partially amassed. This is not to suggest that the experiences of one group always conflated with others, but rather, it is to illustrate that the experiences of the older Canadian men in this study were complementary to each other, despite their heterogenous backgrounds and journeys. The themes were: *Role model choice* and *Processes of roles models facilitating change*. Within the *Role model choice* theme, two subthemes emerged: *Biomedical sphere of perceived ability* and *Negotiation exemplars*.

### 4.1. Theme 1. Role Model Choice: Negotiating “the Realm of [Perceived] Ability”

Participants were able to identify a positive or successful aging role model in their lives. Often, these role models were similar in age to the participants, of the same gender (i.e., male), alive at the time of the interview, and were family members or close acquaintances. Several participants also mentioned role models that were dissimilar in age, different gender (i.e., one mentioned his wife), deceased, and/or high-profile individuals such as celebrities and professional athletes. Overall, conjuring the thought of a personal role model was effortless and ubiquitous, suggesting that the concept of role modelling was at least familiar to these participants, regardless of the value they assigned to various profiles (e.g., celebrity versus acquaintance or a significant other). Additionally, many participants spoke about role models as potential guides (i.e., as valued exemplars or alliance connections) in negotiating age-related challenges (e.g., health conditions) in later life, as exemplified below.

***Biomedical sphere of perceived abilities.*** With regard to salient role model characteristics, there was agreement among participants that the attainment of biomedical criteria of aging success constituted the predominant, albeit not the only, reason for choosing a later-life role model. For example, Neil (76, *inactive*) described his younger brother as his role model:

*Well I have a brother that’s 70 years old that still plays hockey. I feel he’s doing really well…Yeah, he’s a hairdresser. He still works 7 days a week. Yeah, it’s always amazed me the way he can do that. He’s a very young 70.*

This description epitomizes the archetypal role model profile among participants, which is that of a close male acquaintance or family member that is similar in age to the participant, and who is physically, vocationally, and/or socially active on a daily basis. Given the typical proximity and depth of these particular relationships, participants who chose similar role model profiles perceived acquisition of these role models as sensible and pragmatic. In line with Neil’s rationale, Frank (75, *assistance*), mentioned a close friend as his role model: “You know he’s in better shape than I am. [He’s] two years older than I am and he’s still playing [ice hockey]. He’s 78.” Many participants appeared to regard individuals who could play ice hockey into later life as inspiring aging role models most likely because it was a common Canadian pastime that they valued. It is plausible that many of these men, in their role model choice, valued the vicarious continuity of activities that they had participated in across their lives, such as hockey.

Overall, participants in the *active* group tended to have the widest, or most ambitious, spheres of physical, social, and mental abilities, such that the level and extent of functioning and engagement that their role models possessed were greater in absolute terms compared to the other groups. That is, the *active* participants, through their explanations of role models, could foresee themselves obtaining a high level of biomedical ability (or elite transcendence), and they were strongly determined to do so. This notion was particularly obvious during the photo-elicitation session—which, overall, largely bolstered interview findings across groups—perhaps due to the priming that select images of highly active and competitive older men provided. For example, Stephen (78, *active*) selected the image of an older man doing resistance (weight) training as his role model and rationalized his choice: “He’s not just playing a game. He’s improving himself. He’s fending off old age.” Stephen, himself a strength trainer, perceived this role model as within his sphere of potential ability, highlighting the particularly clear vision the active group had towards their goals, and the tenacity with which they intended to reach the perimeter of their sphere where their role models resided. Comparatively, the *inactive* and *assistance* participants tended to negotiate their spheres of ability with slightly less clearly defined and ambitious goals, as expressed through their role model choices described below.

***Negotiation exemplars.*** While it was clear that achieving and/or maintaining biomedical aging success criteria was the perceived ideal in later life, the men in this study, through their role model choices, implied that when this ideal (or elite transcendence) is no longer possible (e.g., due to individual or circumstantial limitations), they are forced to renegotiate their sphere of ability and what they desire in later life. Having a role model who has previously gone through a similar negotiation and who has persevered through difficulties to find meaning in their aging experience may be a useful vicarious strategy for staying actively engaged with life in a manner that is ongoing and important to each individual. For example, Isaac (84, *assistance*), during the photo-elicitation segment, suggested the importance of accepting perceived limitations of aging and indicated that driving, gardening, and playing cards with friends were the images he felt could most represent a personal successful aging role model: “They’re aging gracefully. They’re not trying to do things beyond the realm of ability of someone who is older.” This statement highlights the collective sentiment among all participants, in that they had implicitly or explicitly discerned a realm or sphere of what they themselves, or older adults in general, could realistically achieve or perform in later life. Role models were spoken about as exemplars of how to negotiate this sphere when aging-related obstacles inevitably presented themselves and how to maximize one’s potential within one’s sphere. To further illustrate, William’s (82, *inactive*) role model—his wife—represented successful aging to him because of her resilience through disease and disability:

*[My wife] has had a really rough ride with it [her chronic disease]. Three years now…But she gets through it. I’ve only seen her break down twice. But she just…gets on with it. So, she’s a role model for me…She’s a wonderful lady to be helping because she appreciates it. It makes it worthwhile for me.*

Although a large focus of William’s role model reasoning pertains to perseverance and coping, there is also an underlying emphasis on the importance of avoiding the negative impacts of such age-associated health difficulties. While maintaining biomedical health is important, role models, such as William’s wife, can demonstrate how one might overcome these challenges and perhaps redefine one’s perceived sphere of ability when it becomes unachievable or unrealistic. Chris (77, *active*) also touches on this process of negotiation through his role models:

*Well, I have a couple friends…they’re both turning 80 this spring and they’re both still coaching baseball. I really think that it’s great that two people like them can get out there and still coach and they love it and they keep doing it.*

In stating “two people like them”, Chris perhaps alludes to the sphere of ability that he places on older adults, suggesting that two men in their 80s coaching sports are abutting, or even challenging, what he considers possible in later life. The utility of role models of aging may be, in part, to demonstrate how an older adult may achieve their own unique age-related goals, and it is key to acquire role models that possess relatable, attainable, and perceptively realistic characteristics, as participants often envisioned.

### 4.2. Theme 2. Processes of Role Models Facilitating Change

Most participants expressed the belief that a role model had the potential to influence their aging and health-related attitudes and behaviours. However, the strength and manifestation of this potential influence varied across groups. *Active* group participants typically perceived that their chosen role model implicitly influenced their attitudes and behaviours “by example” or observation (i.e., vicariously). For instance, Alan (80, *active*) mentioned that his busy and active father bestowed an indirect influence on him: “I think he did but…just by watching him I guess. And he didn’t really do anything to really show me the way or anything. Just by example I guess.” Despite this, the extent to which *active* participants emphasized a need for vicarious experience was relatively low compared to other participant groups. This is plausibly tied to their already high level of mastery experience (i.e., knowledge, ability, and motivation) in regular active leisure, in that they do not require help with furthering these levels, but rather a distal comparator for inspiration of what else is possible in their lives. Similarly, James (80, *active*) looked to others for inspiration, particularly among his active friends of a similar age: “It’s encouraging. See if that guy could do it, I’m better than him [*laughing*].” Notably, James alluded to another common *active* group sentiment of innate competition and a constant comparison mechanism by which already active men may be motivated to further increase their physical and social engagement. In essence, many chosen role models appeared to only have the potential to indirectly influence the health-related attitudes and behaviours of *active* participants. Related to this notion is the implication that men in the *active* group envisioned role models as standards (or valued exemplary endeavours) by which they can ensure that they are doing better than the average older man, and when they achieved this form of ‘elite transcendence’, participants generally would be content with their aging experience.

On the other hand, many *inactive* and *assistance* participants required a more direct, explicit, and engaging influence from their role models. For example, Francis’s (78, *inactive*) chosen role model was a close friend with whom he played cards, bowled, and watched horse racing. He spoke to this dynamic influence and that his friend “wants to keep [him] involved” by holding Francis accountable in terms of involvement in such social leisure pursuits. Francis could envision this shared accountability translating to more active leisure, such as exercise. Additionally, William (82, *inactive*) spoke about a strong, direct influence from his wife, his role model, because they “work very closely together.” This was another instance in which a participant perceived that an already established, close relationship could be leveraged and tailored toward achieving aging and physical activity goals through the use of an informal, supportive “buddy system”. During the photo-elicitation, William, in reference to the image depicting a man playing cards with friends, acknowledged the sedentary nature of the activity but further emphasized the importance of companionship in his view on role models:

*You know I actually think, see I like the companionship of this but it’s not exercise. You know what I mean. It’s good to have this…having your friends too, you need that too you know. You need to have somebody to get problems off your chest too…I go in Monday morning, a couple guys all saying this and that, and that’s our ‘get off our chest period’ you know. It’s good to talk about your problems too. Mentally too.*

William highlights the utility of role models in facilitating aging well through an alliance connection that goes beyond that of physical functioning, including cognitive functioning, emotional health, social support, and engagement with life. Largely, it appeared that *inactive* and *assistance* participants needed much more than just an indirect role model to influence their aging-related attitudes and behaviours.

To add complexity to this picture, some *inactive* and *assistance* participants mentioned that they were not influenced by their role models and expressed a sense of disconnect. These instances were also almost always tied to caveats. Edmond, for example, (85, *inactive*) did not appear to be influenced by his role models, who are elite athletes (e.g., ice hockey legend, Gordie Howe), because he was only “just interested in them”. Notably, Edmond was the only *inactive* participant to profile elite athletes as his role models. Perhaps older elite athletes as role models might portray unrealistic standards that are not likely to inspire health-related attitude and behaviour changes in later life. Although high-profile individuals, such as celebrities and elite athletes, were occasionally chosen as potential role models, this was often more from a fascination standpoint rather than a pragmatic or relatable view of what an individual could achieve given their unique abilities and circumstances.

Given that Masters athletes are often held up as exemplars of successful aging in popular press and research (e.g., [68,69,70,71,72]), we sought older men’s opinions on Masters athletes and Masters sport. While participants typically regarded Masters sport as a laudable endeavour, most of them viewed it as inappropriate for their individual circumstances, nor realistically influential in personal health-related attitude and behaviour changes. For example, Joe (79, *inactive*) expressed that health limitations and risks are a barrier for his involvement in Masters sport, but he also recognized that some competitive sports may be safer than others:

*I like it, it’s exercise, it depends on what exercise you’re doing. Older folks, they have to be careful with what they’re doing at all times…they’re subject to getting a heart attack quicker than just anybody…it all depends on what you’re doing…There’s different things you can do and enjoy.*

In reference to competitive Masters sport, Stephen (78, *active*), a non-competitive strength trainer, said: “I feel that the sport that I am doing is sufficient to keep me healthy. Now, like you can get hurt like playing a sport and then I can’t work out! What’s that do? That’s stupid.” Stephen’s lament is intriguing in that, to him, competing in elite sport is not only deemed unrealistic for many in later life, but an unnecessary risk to one’s physical health.

This sentiment of high-level sport as inherently and disproportionately risky, and potentially life-altering or life-threatening, compared to non-competitive active leisure or exercise, was widespread among our participants, suggesting possible perceived limitations of Masters sport and high-level Masters athlete role models as realistic in facilitating increased levels of physical activity, exercise, or sport involvement among older men. For instance, Neil (76, *inactive*) could appreciate Masters sport, but the competitiveness discouraged his participation: “I see nothing wrong with it. But then again, I can’t take it that serious. You know, winning at any cost like some people think…And I think I’ve lost a lot of the competitive spirit.” These novel findings have implications for health promoters and exercise rehabilitation specialists working with older men and role models.

## 5. Discussion

### 5.1. Exemplars of Biomedical Aging Success

Our study explored the perspectives of older men on role models of aging success and determined strategies for how role models can affect changes in their perceptions and practices associated with sport, exercise, health, and aging. All participants were able to envision one or several potential role models in their lives who could influence their aging-related attitudes and behaviours, which both supported and extended previous research on this topic [12,13]. Perhaps most notably was the emphasis that participants placed on the biomedically based characteristics and achievements of their aging role models. Specifically, participants tended to choose role models who, in later life, avoided disease and disability, stayed actively engaged with social and physical pursuits, and maintained high levels of physical and cognitive functioning. These characteristics essentially represented the biomedical criteria of aging success within Rowe and Kahn’s [23] model. Our observations are consistent with that of Jopp et al. [13], who also found that biomedical criteria were most salient in role model selection among older adults. This may, in part, reflect the pervasive medicalized view of aging held by many individuals and groups within Western societies [21], in which aging is viewed largely as a process of physiological deterioration that must be mitigated by intervention, particularly in sport and exercise sciences [19,73]. Thus, procuring and promulgating this biomedically based successful aging role model profile may resonate most with older men, at least topically.

Nevertheless, a key consideration in our findings and that of Jopp et al.’s [13] is that, while biomedical criteria were most frequently discussed in both studies, many other characteristics of aging success were also mentioned, such as attitudes toward life, meaning in life, and resilience. Our previous work also supports this notion that biomedically aging success tends to be important to many older adults, but many also value additional, alternative criteria in their personal aging experiences, such as solitude and passive pursuits [18,33]. Moreover, by not overly relying on the medicalized aspect of exemplars of aging success, there is opportunity for fewer psychological and emotional challenges when the inevitable biomedical processes associated with aging manifest [20]. Participants seemed to support this notion with their view of Masters athletes as unrealistic role models, which poses a challenge to claims that Masters athletes are exemplars of aging success found in academic literature and popular press (e.g., [68,69,70,71,72]). As argued by Dionigi and Gard [25] and Gard et al. [21], the concept of Masters athletes as exemplars of successful aging is grounded on problematic ‘sport for all’ policies based on highly individualised assumptions about self-responsibility for health, which ignore social determinants of health and aging, demonise passive leisure, and disregard multiple ways of aging well.

Therefore, in helping an older adult acquire a role model or when selecting a role model for portrayal in popular press and health promotion, it may be useful to highlight not only achievements related to the physical and cognitive health of that role model, but also their positive attitude toward aging. A positive self-perception toward aging obtained vicariously may ultimately lead to beneficial self-fulfilling prophecies such as increased engagement in social and active leisure, as consistent with stereotype embodiment theory [47] and Bandura’s self-efficacy theory [38] and as reported by the men in the current study.

### 5.2. Older Men and Role Models: Strategies for Facilitating Change

The noted variations in role model profiles across the men and activity groups also indicate that perhaps these men could envision relying on a range of role models to facilitate personal aging success, and that this may ultimately depend on individual preferences, stage of life, and role model availability. Moreover, a pattern emerged in which many participants stated or implied a perceived sphere of what is possible for them in later life (e.g., from a physical, cognitive, and/or social standpoint), and most role models evoked were within this ‘sphere of perceived ability’. Theme 1 (Role model choice: Negotiating “the realm of [perceived] ability”) showed that the active group had a larger perspective with respect to the realm of ability but was more focused on a biomedical approach when compared to the inactive and assistance groups. Overall, the findings indicated the importance of *realistic and attainable* role models, based on the perceived realm of ability that these older adults have at a particular point in their lives, which may change over time. Theme 2 showed that there were various processes of role models facilitating change across the three groups of men.

Accordingly, we propose four key strategies in which role models can facilitate change in perceptions and behaviours based on our interpretation of results. First, role models may influence older men through the negotiation process of *elite transcendence*, in which an elite (e.g., highly active or successful) role model facilitates an older adult’s ambition to achieve aging-related goals that transcend what an average older adult is able to accomplish in similar circumstances. This transcendence may also be related to the expansion of one’s perceived sphere of abilities. To illustrate, a couple of the men (Neil and Frank) described how they were amazed and felt inspired by their very active role models who were still playing hockey in their 70s. Overall, very few participants in our study expressed a desire to transcend to elite levels of sport through competitive athlete role model choices. This is consistent with previous findings that ‘superstar’ role models can actually backfire in terms of inspiring change because they are unrealistic and unattainable for most [74]. It is also possible that this category involves older adults who already believe they are at elite (biomedical) levels and that there is likely no possible role model for their level of self-described successful aging. However, this categorization is complicated by previous work that has observed that some older adults, who are regularly active but not yet necessarily at elite levels of engagement, may be influenced toward particularly high achievement by a role model [11].

Secondly, role models provided many older men with a *valued exemplary endeavour*. These men expressed the potential to be influenced by a role model up to a certain and suitable gratifying goal, but beyond that point, a role model has little influence on them. That is, these participants emphasized the need for a realistic and attainable role model that could help them thrive within their spheres of perceived ability, but that transcending their spheres would be unnecessary or potentially harmful. For example, Stephen (*active*) selected a man lifting weights as a realistic potential role model during the photo-elicitation, but also discussed in the interview how high-level competitive sport beyond a controlled workout would be dangerous to his health.

Thirdly, participants described the process of *alliance connection*, in which the role model tended to be a significant other, and it was about the interactions and mutually strived for goals between the older man and their role model, which also involved the men striving for realistic goals within one’s sphere, but in which there was a greater emphasis on mutual achievement and social and emotional support. These individuals appeared content with marginal progress toward aging-related goals because what seemed most important was that they were striving together with their role model. These relationships tended to be tightly knit, such as William’s (*inactive*) life partnership with his wife. Similarly, Jopp et al. [13] speculated that having an in-depth, close connection with a family member role model may provide a more proximal, realistic, elaborate, and directly engaging exemplar of what is possible in one’s later life. Accordingly, a plausible health promotion strategy for helping older adults achieve their goals and increase levels of exercise or active leisure engagement may include identifying a close individual in one’s life, if available, and encouraging a role model partnership based on pragmatic and meaningful, mutual achievements.

A fourth strategy, in which the role modelling process among our participants was negotiated, we labelled *disconnect and caveats*. This category involved participants who did not envision a role model as being beneficial or influential for various reasons. These participants often mentioned high-profile role models such as celebrities or athletes who they deemed out of reach. This finding reiterates the importance of determining “realistic” role models for older men, that is, role models that are simultaneously individualized with the freedom to change as the person ages and experiences their life.

When applying these four perspectives in practice, health promotion efforts could aim to identify which process older adults perceive role models can facilitate change in their perceptions, actions, and capabilities, and then match role models appropriately or, in the case of the *disconnect and caveats* perspective, assist older adults in finding a more realistic role model who could inspire change. Ultimately, we illuminated the potential heterogenous strategies in which role models may be used by older men in negotiating their perceptions and behaviours related to aging, exercise, and active leisure, establishing direction for future research in this area that has received relatively little attention.

### 5.3. Role Models, Older Adults and Gender Differences

The findings in this study shared similarities and differences with Horton et al.’s [11] investigation of older women’s aging role models. Men and women across both studies often chose close personal acquaintances or family members as their role models. That is, chosen role models were typically similar in age and gender and were engaged in slightly greater, but perceptively attainable, levels of physical, social, and cognitive pursuits than the participant. When asked about their opinions on Masters sport, many participants in both studies tended to view Masters athletes’ accomplishments as laudable but unrealistic or even dangerous for them.

However, in contrast to Horton et al.’s ([11], p. 39) finding among older women that “virtually all participants described individuals who were personal acquaintances,” a notable minority of older men in the current study selected high-profile athletes or celebrities as role models (e.g., Gordie Howe). Thus, the role modelling process may be based on gender preferences and socio-historical contexts. For example, the early-life formative experiences of most of the older people, in both this study and Horton et al.’s [11] study, coincided with an era in which socially constructed norms positioned sport and physical activity as more appropriate for males than females, and in which the former were traditionally expected to participate in more competitive activities both physically and vocationally [75,76].

Traditionally, men were believed to have a more instrumental work-related role in society versus that of a woman’s more socioemotional role focused on marriage and family (e.g., [76,77]). Moreover, it has been suggested that masculine ideals can result in a greater predilection, compared to women, toward active leisure that is competitive and sport-based, although these are largely generalities [14,75]. In the current study, a common *active* group sentiment was that of role models providing them with innate competition and a constant comparison mechanism by which, as already active men, they could further increase their physical and social engagement. Similar to findings on older male athletes [78], social comparisons were made by the older men in the current study to separate themselves from others, thereby cultivating a positive perception of their own aging and current capabilities.

These cultural stereotypes and gender norms may help to explain the role model choices and reasonings of the older men in the current study who responded more favourably, compared to the older women in Horton et al.’s [11] study, to role model profiles of high-level athletes/celebrities. However, the current findings also revealed that the overall effectiveness of such a strategy in terms of fostering attitude and behaviour changes in older men is unclear and requires further research. Participants in our study who chose these role models also implied that, while their role models’ achievements were commendable, the actual influence on participants’ lives were marginal. From a health promotion standpoint, it makes sense to endorse role model profiles that are not too rigidly based on traditionally perceived masculine ideals of success in terms of elite athleticism. Previous findings from our work [9,18,78] and that of others (e.g., [79]) suggests a disparity between societal masculine norms, such as self-reliance, strength, and stoicism, and what older men actually prefer in their leisure, such as the men in the current study who discussed role models that provide emotional and social support. This outcome reveals that alternative avenues of expressing oneself through sport, exercise, or leisure, which may emphasize companionship and enjoyment over independence and competition, for example, could resonate with more men and inspire change among older men.

## 6. Limitations and Future Directions

While this study examined perceptions of the role modelling processes among a range of older men, it is limited in its generalizability given the predominately white, middle-class, and community-dwelling sample. Future research should explore the role modelling process among older adults of varying demographic profiles to broaden our understanding across cultural and socioeconomic groups. Moreover, while we aimed to bolster our data with a pluralistic methodology, our approach was limited in temporality such that our traditional interviews and photo-elicitation segment occurred in the same session. However, we found many instances in which participants’ responses in the photo-elicitation added valuable nuance that would not have been uncovered with one method alone.

## 7. Conclusions

The older Canadian men sampled in our study expressed a range of choices in their envisioned role models of aging success. Their reasoning also varied, although choosing a role model for their biomedical aging-related attainments was most predominant. Most participants discussed a desire for realistic and pragmatic role models, suited to their preferences and perceived abilities. High-achieving celebrity and athlete role models were lauded by some of the men, but they were typically viewed as unrealistic and not likely to inspire behaviour or attitude changes. When designing initiatives, health promoters and exercise rehabilitation specialists need to consider the heterogeneity in the role modelling processes and strategies for facilitating change among older men. It appears salient, when feasible, to encourage role models, passively or actively, that are tailored to an individual’s views on aging, exercise, and active leisure, rather than those based on masculine stereotypes of competitiveness, wealth, individualism, and physical strength.

Ultimately, role models of aging success may be an underutilized and understudied phenomenon, given the sentiment among our participants that role models could provide enjoyment, meaning, and benefit toward attitude and behaviour change in later life. On a societal level, while promoting biomedical achievements of role models remains important and will likely resonate with many older men, when applied too closely, there may be potential for overmedicalization that could miss an opportunity to capitalize on uncovering the latent and less overt importance that older men place on aspects beyond the biomedical realm of aging and traditional masculine ideals.

## Figures and Tables

**Table 1:** Participant Details ^†^.

Physical Activity Group	Pseudonym	Age	Education	Past Profession	Marital Status
Active (n = 6)	Alan	80	One year of university	Data processor	Married
	Albert	79	Doctorate	Professor/piano instructor *	Married
	Chris	77	Teacher’s college	Teacher	Married
	Edwin	80	Master’s degree	Principal	Married
	James	80	Teacher’s college	Teacher	Widower
	Stephen	78	One year of university	Personal trainer **	Married
Inactive (n = 6)	Edmond	85	Grade 11	Photo editor	Widower
	Francis	78	Trade school	Mechanic	Married
	Joe	79	Grade 11	Business owner	Married
	Neil	76	Grade 12	Production worker	Married
	Russell	76	Business School	Production worker	Married
	William	82	High School	Tool maker	Married
Active with Assistance (n = 7)	Carl Frank	78 75	Master’s degree Grade 12	Sales Estimator	Married Married
	Fred	78	Middle school	Production worker	Single/never married
	George	85	Grade 11	Sales	Married
	Isaac	84	Post-secondary	Journalist	Married
	John	75	Grade 10	Engineer	Married
	Robert	90	Grade school	Transport	Married

Notes. * Still working full-time. ** Still working part-time. ^†^ This is a part of a Masters thesis by Jordan Deneau, University of Windsor, Canada.

## Data Availability

The data presented in this study are available on request from the corresponding author. The data are not publicly available due to the qualitative nature of the study.

## References

[1] Statistics Canada (2019). Population Projections: Canada, Provinces and Territories, 2018 to 2068. https://www150.statcan.gc.ca/n1/daily-quotidien/190917/dq190917b-eng.htm.

[2] Statistics Canada (2021). Physical Activity, Self Reported, Adult, by Age Group. https://www150.statcan.gc.ca/t1/tbl1/en/tv.action?pid=1310009613.

[3] Sallis J.F., Bull F., Guthold R., Heath G.W., Inoue S., Kelly P., Oyeyemi A.L., Perez L.G., Richards J., Hallal P.C. (2016). Progress in physical activity over the Olympic quadrennium. Lancet.

[4] United Nations (2017). World Population Prospects 2017 Revision. https://esa.un.org/unpd/wpp/Publications/Files/WPP2017_KeyFindings.pdf.

[5] Chodzko-Zajko W.J., Proctor D.N., Fiatarone Singh M.A., Minson C.T., Nigg C.R., Salem G.J., Skinner J.S. (2009). American College of Sports Medicine position stand. Exercise and Physical Activity for Older Adults. Med. Sci. Sports Exerc..

[6] Gayman A.M., Fraser-Thomas J., Dionigi R.A., Horton S., Baker J. (2017). Is sport good for older adults? A systematic review of psychosocial outcomes of older adults’ sport participation. Int. Rev. Sport Exerc. Psychol..

[7] del Barrio E., Marsillas S., Buffel T., Smetcoren A.-S., Sancho M. (2018). From Active Aging to Active Citizenship: The Role of (Age) Friendliness. Soc. Sci..

[8] Costello E., Kafchinski M., Vrazel J., Sullivan P. (2011). Motivators, Barriers, and Beliefs Regarding Physical Activity in an Older Adult Population. J. Geriatr. Phys. Ther..

[9] Deneau J., Horton S., van Wyk P.M. (2020). Seven A’s of Active Aging: Older Men’s Suggestions for Physical Activity Programs. J. Aging Phys. Act..

[10] Rasinaho M., Hirvensalo M., Leinonen R., Lintunen T., Rantanen T. (2007). Motives for and Barriers to Physical Activity among Older Adults with Mobility Limitations. J. Aging Phys. Act..

[11] Horton S., Dionigi R.A., Bellamy J. (2013). Canadian women aged 75 and over: Attitudes towards health related role models and female masters athletes. Int. J. Interdiscip. Soc. Community Stud..

[12] Horton S., Baker J., Côté J., Deakin J.M. (2008). Understanding Seniors’ Perceptions and Stereotypes of Aging. Educ. Gerontol..

[13] Jopp D.S., Jung S., Damarin A.K., Mirpuri S., Spini D. (2016). Who Is Your Successful Aging Role Model?. J. Gerontol. Ser. B.

[14] Bottorff J.L., Seaton C.L., Johnson S.T., Caperchione C.M., Oliffe J.L., More K., Jaffer-Hirji H., Tillotson S.M. (2014). An Updated Review of Interventions that Include Promotion of Physical Activity for Adult Men. Sports Med..

[15] Carroll P., Kirwan L., Lambe B. (2014). Engaging ‘hard to reach’ men in community based health promotions. Int. J. Health Promot. Educ..

[16] Deneau J., Dionigi R.A., Horton S. (2020). The Benefits of Masters Sport to Healthy Aging. Sport Information Resource Centre (SIRC). https://sirc.ca/blog/the-benefits-of-masters-sport-to-healthy-aging/.

[17] Smith J., Braunack-Mayer A., Wittert G., Warin M. (2007). “I’ve been independent for so damn long!”: Independence, masculinity and aging in a help seeking context. J. Aging Stud..

[18] Deneau J., van Wyk P.M., Horton S. (2019). Capitalizing on a “Huge Resource”: Successful Aging and Physically Active Leisure Perspectives from Older Males. Leis. Sci..

[19] Tulle E. (2008). Ageing, the Body and Social Change: Running in Later Life.

[20] Dionigi R.A. (2017). I Would Rather Die Than Live Sedentary. Top. Geriatr. Rehabil..

[21] Gard M., Dionigi R.A., Dionigi C., Dionigi R., Gard M. (2018). From a Lucky Few to the Reluctant Many: Interrogating the Politics of Sport for All. Sport and Physical Activity Across the Lifespan: Critical Perspectives.

[22] Martinson D.M., Berridge M.C. (2014). Successful Aging and Its Discontents: A Systematic Review of the Social Gerontology Literature. Gerontol..

[23] Rowe J.W., Kahn R.L. (1997). Successful Aging. Gerontologist.

[24] Gard M., Dionigi R.A., Horton S., Baker J., Weir P., Dionigi C. (2017). The normalization of sport for older people?. Ann. Leis. Res..

[25] Dionigi R., Gard M., Dionigi R., Gard M. (2018). Sport for all ages: Weighing the evidence. Sport and Physical Activity across the Lifespan: Critical Perspectives.

[26] Gard M., Dionigi R.A. (2016). The world turned upside down: Sport, policy and ageing. Int. J. Sport Policy Politics.

[27] Son J., Dionigi R.A., Kono S., Spracklen K., Beniwal A., Sharma P. (2020). The complexity of sport-as-leisure in later life. Positive Sociology of Leisure.

[28] Geard D., Reaburn P., Rebar A., Dionigi R. (2017). Masters Athletes: Exemplars of Successful Aging?. J. Aging Phys. Act..

[29] Higgs P., Gilleard C., Tulle E., Phoenix C. (2015). Fitness and Consumerism in Later Life. Physical Activity and Sport in Later Life.

[30] Jeste D.V., Depp C.A., Vahia I.V. (2010). Successful cognitive and emotional aging. World Psychiatry.

[31] Jopp D.S., Wozniak D., Damarin A.K., De Feo M., Jung S., Jeswani S. (2015). How Could Lay Perspectives on Successful Aging Complement Scientific Theory? Findings from a U.S. and a German Life-Span Sample. Gerontol..

[32] Phelan E.A., Anderson L.A., Lacroix A.Z., Larson E.B. (2004). Older adults’ views of “successful aging”—How do they compare with researchers’ definitions?. J. Am. Geriatr. Soc..

[33] Dionigi R.A., Horton S., Bellamy J. (2011). Meanings of Aging Among Older Canadian Women of Varying Physical Activity Levels. Leis. Sci..

[34] Dionigi R.A. (2016). The competitive older athlete: A review of psychosocial and sociological issues. Top. Geriatr. Rehabil..

[35] Ory M., Hoffman M.K., Hawkins M., Sanner B., Mockenhaupt R. (2003). Challenging aging stereotypesStrategies for creating a more active society. Am. J. Prev. Med..

[36] Bandura A. (1977). Social Learning Theory.

[37] Bandura A. (1986). Social Foundations of Thought and Action: A Social Cognitive Theory.

[38] Bandura A. (1986). The Explanatory and Predictive Scope of Self-Efficacy Theory. J. Soc. Clin. Psychol..

[39] Dionigi R. (2007). Resistance Training and Older Adults’ Beliefs about Psychological Benefits: The Importance of Self-Efficacy and Social Interaction. J. Sport Exerc. Psychol..

[40] Dionigi R.A., Fields Z.T. (2016). Resistance training in later life: Self-efficacy, physical self-worth and sense of community. Resistance Training: Principles, Adaptations and Health Effects.

[41] Morgenroth T., Ryan M.K., Peters K. (2015). The Motivational Theory of Role Modeling: How Role Models Influence Role Aspirants’ Goals. Rev. Gen. Psychol..

[42] Zirkel S. (2002). Is there a place for me? Role models and academic identity among white students and students of color. Teach. Coll. Rec..

[43] Chlosta S., Patzelt H., Klein S.B., Dormann C. (2010). Parental role models and the decision to become self-employed: The moderating effect of personality. Small Bus. Econ..

[44] Chen E., Lee W.K., Cavey L., Ho A. (2012). Role Models and the Psychological Characteristics That Buffer Low-Socioeconomic-Status Youth from Cardiovascular Risk. Child Dev..

[45] Levy B.R., Banaji M.R., Nelson T.D. (2004). Implicit ageism. Ageism: Stereotyping and Prejudice against Older Persons.

[46] Sarkisian C.A., Prochaska T.R., Wong M.D., Hirsh S.A., Mangione C.M. (2005). The relationship between expectations for aging and physical activity among older adults. J. Gen. Intern. Med..

[47] Levy B.R. (2009). Stereotype Embodiment: A Psychological Approach to Aging. Curr. Dir. Psychol. Sci..

[48] Dionigi R.A. (2015). Stereotypes of Aging: Their Effects on the Health of Older Adults. J. Geriatr..

[49] Higgins E.T. (1998). Promotion and Prevention: Regulatory Focus as A Motivational Principle. Adv. Exp. Soc. Psychol..

[50] Lockwood P., Chasteen A.L., Wong C. (2005). Age and Regulatory Focus Determine Preferences for Health-Related Role Models. Psychol. Aging.

[51] Francis A. (2019). Carol Lafayette-Boyd May be the Most Modest WMA Athlete of the Year Ever. Canadian Running. https://runningmagazine.ca/the-scene/carol-lafayette-boyd-may-be-the-most-modest-wma-athlete-of-the-year-ever/.

[52] Horton S., Baker J., Horton S., Weir P.L. (2010). Masters athletes as role models? Combating stereotypes of aging. The Masters Athlete: Understanding the Role of Sport and Exercise in Optimizing Aging.

[53] Kite M.E., Stockdale G.D., Whitley B.E., Johnson B.T. (2005). Attitudes Toward Younger and Older Adults: An Updated Meta-Analytic Review. J. Soc. Issues.

[54] Dionigi R.A. (2006). Competitive sport and aging: The need for qualitative sociological research. J. Aging Phys. Act..

[55] Denzin N.K., Lincoln Y.S., Denzin N.K., Lincoln Y.S. (2008). Introduction: The discipline and practice of qualitative research. The Landscape of Qualitative Research.

[56] Wojnar D.M., Swanson K.M. (2007). Phenomenology. J. Holist. Nurs..

[57] Merriam S.B. (2002). Qualitative Research in Practice: Examples for Discussion and Analysis.

[58] Patton M.Q. (2015). Qualitative Research and Evaluation Methods.

[59] Chamberlain K., Cain T., Sheridan J., Dupuis A. (2011). Pluralisms in Qualitative Research: From Multiple Methods to Integrated Methods. Qual. Res. Psychol..

[60] Williams T.L. (2018). Exploring narratives of physical activity and disability over time: A novel integrated qualitative methods approach. Psychol. Sport Exerc..

[61] Tremblay M.S., Warburton D.E., Janssen I., Paterson D.H., Latimer A.E., Rhodes R.E., Kho M.E., Hicks A., LeBlanc A.G., Zehr L. (2011). New Canadian Physical Activity Guidelines. Appl. Physiol. Nutr. Metab..

[62] Creswell J.W. (2014). Research Design: Qualitative, Quantitative, and Mixed Methods Approaches.

[63] Bigante E. (2010). The Use of Photo-Elicitation in Field Research. http://echogeo.revues.org/11622.

[64] Thomas M.E. (2009). Auto-Photography.

[65] Harper D. (2002). Talking about pictures: A case for photo elicitation. Vis. Stud..

[66] Braun V., Clarke V. (2019). Reflecting on reflexive thematic analysis. Qual. Res. Sport Exerc. Health.

[67] Corbin J., Strauss A. (2008). Basics of Qualitative Research: Techniques and Procedures for Developing Grounded Theory.

[68] Cooper L.W., Powell A.P., Rasch J. (2007). Master’s swimming: An example of successful aging in competitive sport. Curr. Sports Med. Rep..

[69] Geard D., Rebar A.L., Dionigi R.A., Reaburn P.R.J. (2021). Testing a Model of Successful Aging on Masters Athletes and Non-Sporting Adults. Res. Q. Exerc. Sport.

[70] Geard D., Rebar A.L., Dionigi R., Rathbone E., Reaburn P. (2021). Effects of a 12-Week Cycling Intervention on Successful Aging Measures in Mid-Aged Adults. Res. Q. Exerc. Sport.

[71] Hawkins S.A., Wiswell R.A., Marcell T.J. (2003). Exercise and the Master Athlete—A Model of Successful Aging?. J. Gerontol. Ser. A Biol. Med. Sci..

[72] Louis J., Nosaka K., Brisswalter J. (2012). The endurance master athlete: A model of successful ageing. Sci. Sports.

[73] Phoenix C., Tulle E., Piggin J., Mansfield L., Weed M. (2018). Physical activity and ageing. Routledge Handbook of Physical Activity Policy and Practice.

[74] Lockwood P., Kunda Z. (1997). Superstars and me: Predicting the impact of role models on the self. J. Pers. Soc. Psychol..

[75] Pfister G. (2012). It is never too late to win—Sporting activities and performances of ageing women. Sport Soc. Cult. Commer. Media Politics.

[76] Vertinsky P.A. (1995). Stereotypes of Aging Women and Exercise: A Historical Perspective. J. Aging Phys. Act..

[77] Cumming E., Henry W.E. (1961). Growing Old: The Process of Disengagement.

[78] Horton S., Dionigi R., Gard M., Baker J., Weir P., Deneau J. (2019). “You Can Sit in the Middle or Be One of the Outliers”: Older Male Athletes and the Complexities of Social Comparison. Front. Psychol..

[79] Thandi M.K.G., Phinney A., Oliffe J.L., Wong S., McKay H., Gould J.S., Sahota S. (2018). Engaging Older Men in Physical Activity: Implications for Health Promotion Practice. Am. J. Men’s Health.

